# GC-MS Profiling and In Vitro Assessment of Antimicrobial, Antioxidant, and Anti-Inflammatory Activities of Essential Oils from Wild-Growing *Glycosmis lanceolata* (Blume) D. Dietr. in Vietnam

**DOI:** 10.3390/molecules31132246

**Published:** 2026-06-25

**Authors:** Quang Vuong Le, Ha Thi Thu Chu, Thuy Thi Thu Dinh, Thi Minh Chau Dao, Thi Huyen Thai, Thi Nghiem Vu, Ha Chi Vuong, William N. Setzer

**Affiliations:** 1 Faculty of Biology, Vinh University, 182 Le Duan Street, Truong Vinh Ward, Nghe An 43000, Vietnam; vuong201173@vinhuni.edu.vn (Q.V.L.); daochau27@gmail.com (T.M.C.D.); 2 Institute of Biology, Vietnam Academy of Science and Technology (VAST), 18 Hoang Quoc Viet, Nghia Do, Hanoi 10072, Vietnam; 3 Institute of Chemistry, Vietnam Academy of Science and Technology (VAST), 18 Hoang Quoc Viet, Nghia Do, Hanoi 10072, Vietnam; dttthuy@ich.vast.vn; 4 Faculty of Agronomy, Hue University of Agriculture and Forestry (HUAF), 102 Phung Hung, Hue City 49000, Vietnam; thaihuyen@hueuni.edu.vn; 5 Institute of Materials Science, Vietnam Academy of Science and Technology (VAST), 18 Hoang Quoc Viet, Nghia Do, Hanoi 10072, Vietnam; vtnghiem@ims.vast.ac.vn; 6 Newton Grammar School, 136 Ho Tung Mau, Phu Dien, Hanoi 10053, Vietnam; vuonghachi2203@gmail.com; 7 Aromatic Plant Research Center, 230 N 1200 E, Suite 100, Lehi, UT 84043, USA; setzerw@uah.edu; 8 Department of Chemistry, University of Alabama in Huntsville, Huntsville, AL 35899, USA

**Keywords:** bioactivities, *β*-bisabolene, brevifolin, (*E*)-*β*-caryophyllene, essential oil composition, *Glycosmis lanceolata*

## Abstract

This study evaluates the chemical composition and bioactivities of essential oils extracted from the leaves and twigs of *Glycosmis lanceolata* growing in a natural forest in Vietnam. gas chromatography–mass spectrometry identified 42 and 43 constituents in the leaf and twig oils, respectively. The main compounds in the leaf oil were (*E*)-*β*-caryophyllene (10.2%), *β*-bisabolene (23.7%), and brevifolin (21.3%), while the twig oil was dominated by *β*-bisabolene (11.6%) and brevifolin (12.7%). Neither oil exhibited inhibitory effects against two beneficial bacterial strains, *Bacillus subtilis* and *Lactobacillus fermentum*. In contrast, both oils showed weak antimicrobial activity against four pathogenic bacteria—*Staphylococcus aureus*, *Salmonella enterica*, *Escherichia coli*, and *Pseudomonas aeruginosa*—and one yeast, *Candida albicans*, with IC_50_ values ranging from 2012 ± 118 to 10,593 ± 557 µg/mL. Notably, the twig oil demonstrated pronounced anti-inflammatory activity via inhibition of nitric oxide production (IC_50_ = 29.7 ± 2.58 µg/mL), whereas the leaf oil showed no detectable activity within the tested concentrations. Similarly, DPPH radical scavenging assays indicated stronger antioxidant activity for the twig oil compared to the leaf oil. These findings provide new insights into the phytochemistry and bioactivities of *G. lanceolata* essential oils and may support further investigations into their potential applications.

## 1. Introduction

The genus *Glycosmis*, belonging to the Rutaceae family, comprises tropical plants that are rich in diverse secondary metabolites with notable phytochemical and pharmacological potential. Major bioactive constituents identified in *Glycosmis* species include alkaloids, flavonoids, terpenoids, phenolics, and sulfur-containing amides, as consistently reported in the literature [1,2,3,4,5,6]. Extracts from this genus have been shown to exhibit a wide range of biological activities, including hepatoprotective, antioxidant, antidiabetic, anti-arthritic, antimicrobial, antiviral, and anticancer effects [7,8,9,10]. In Vietnam, several *Glycosmis* species have also been investigated, with reported cytotoxic activity of *G. parviflora* against selected cell lines [11], anticancer potential of *G. ovoidea* [12], and antidiabetic activity of *G. pentaphylla* [13]. In traditional medicine, *Glycosmis* species are widely used for the treatment of fever, infections, liver disorders, digestive ailments, and skin diseases [14]. Collectively, these findings highlight the considerable potential of this genus for applications in both traditional and modern medicine.

In recent years, increasing attention has been directed toward the characterization of essential oils from *Glycosmis* species to better understand their potential applications in natural medicine and cosmetics. Among them, *G. pentaphylla* is the most extensively investigated and has attracted considerable scientific interest. For instance, the chemical composition of essential oils obtained from the bark, leaves, and seeds of *G. pentaphylla* collected in India has been reported, with major constituents including 2-undecanone, 2-tridecanone, 6,10,14-trimethyl-2-pentadecanone, hexadecanoic (palmitic) acid, linalool, and terpinen-4-ol, depending on the plant part [15]. In addition, several studies have further characterized the chemical profile of leaf essential oil from this species [16,17]. The chemical composition of essential oils from other *Glycosmis* species has also been documented, including *G. lucida* [18] and *G. parviflora* [19]. In Vietnam, research has likewise explored essential oils from several *Glycosmis* species, such as the chemical composition of *G. mauritiana* [20] and *G. pentaphylla* [21], as well as the chemical composition and herbicidal activity of *G. puberula* var. *eberhardtii* [22].

*Glycosmis lanceolata* is a shrub or small tree predominantly distributed in humid tropical forests of Southeast Asia. It is characterized by lanceolate leaves, small white flowers, and red–orange berries at maturity [23]. Although less extensively studied than other species within the genus, *G. lanceolata* is regarded as a promising source for phytochemical investigation and natural product discovery. Previous studies on samples collected in China (syn. *G. montana*) have identified a range of non-volatile secondary metabolites, including sulfur-containing flavanols and flavanol dimers from aerial parts (twigs and leaves) [24], as well as indole and carbazole alkaloids exhibiting notable biological activities such as anti-HIV and cytotoxic effects [25]. Additional compounds have been reported to possess cytotoxic activity against liver and colorectal cancer cell lines, along with antimicrobial properties [26]. More recently, a series of acridone alkaloids with cytotoxic activity against the acute lymphoblastic leukemia B-cell line NALM-6 was isolated from branch extracts of this species [27]. To date, only one study has reported the chemical composition of essential oil from *G. lanceolata* leaves collected in Da Nang, Vietnam, identifying limonene (59.3%) as the predominant component [28]. However, that study did not provide retention index (RI) values for the identified compounds, limiting the reliability and comparability of compound identification.

Overall, existing studies indicate that the genus *Glycosmis* represents a rich source of both volatile and non-volatile bioactive compounds, with considerable potential for applications in pharmaceuticals, functional foods, and cosmetics. However, investigations of essential oils within this genus remain limited, and many species, including *G. lanceolata*, are still underexplored. In particular, studies addressing the antimicrobial and anti-inflammatory activities of *Glycosmis* essential oils are scarce, while antioxidant activity has been reported for only a single species, *G. pentaphylla* [17].

Therefore, further research on the chemical composition and biological properties of less-studied species is essential to advance our understanding of this genus and to uncover new application opportunities. To the best of our knowledge, this study provides the first comprehensive evaluation of both the chemical composition and the antimicrobial, anti-inflammatory, and antioxidant activities of essential oils derived from the leaves and twigs of *G. lanceolata*. These findings contribute to the more effective utilization of this species and support its potential applications in medicine, biotechnology, and sustainable development.

## 2. Results

### 2.1. Composition Profiling of Glycosmis lanceolata Leaf and Twig Essential Oils

The essential oils extracted from the leaves and twigs of the *Glycosmis lanceolata* were pale yellow in color and exhibited partial crystallization at temperatures below 25 °C. The essential oil yields were 0.201% and 0.064% (*w*/*w*), calculated based on the respective dried leaf and twig weights. Chemical analysis by GC–MS led to the identification of 42 and 43 compounds, accounting for 91.1% and 88.0% of the total oil compositions, respectively. The predominant chemical groups in the leaf and twig oils were sesquiterpene hydrocarbons (49.5% and 22.5%), oxygenated sesquiterpenes (12.7% and 23.5%), and benzenoids (25.5% and 30.7%). Three major constituents identified in the leaf and twig oils were: (*E*)-*β*-caryophyllene (10.2% and 5.7%), *β*-bisabolene (23.7% and 11.6%), and brevifolin (21.3% and 12.7%). All of these main compounds occurred at markedly higher concentrations in the leaf essential oil. In contrast, several other relatively abundant constituents were detected at considerably lower concentrations in the leaf oil than the twig oil, including linalool (0.5 and 7.9%), methyleugenol (2.5 and 9.3%), methyl *N*-methylanthranilate (trace and 5.3%), and caryophyllene oxide (3.0 and 6.6%). The remaining compounds were present in concentrations ranging from trace amounts (Tr) to 3.9% of the total oils (Table 1).

Although the qualitative compositions of the two oils were generally similar, variations in the relative abundance of individual constituents were observed, which may contribute to differences in their biological activities and physicochemical properties. Chromatograms of essential oils from leaves and from twigs of wild-growing *Glycosmis lanceolata* in Vietnam can be found in section: Supplementary Materials.

### 2.2. Antimicrobial Activity of Glycosmis lanceolata Leaf and Twig Essential Oils

The antimicrobial activity of essential oils extracted from the leaves and twigs of *G. lanceolata* was evaluated against seven microbial strains, including three Gram-positive bacteria (S*taphylococcus aureus*, *Bacillus subtilis*, and *Lactobacillus fermentum*), three Gram-negative bacteria (*Salmonella enterica*, *Escherichia coli*, and *Pseudomonas aeruginosa*), and one yeast strain (*Candida albicans*). In general, the *G. lanceolata* leaf and twig essential oils showed no inhibitory effect against *B. subtilis* and *L. fermentum* at the test concentrations. Among the five pathogenic microbial strains tested, the twig essential oil exhibited stronger inhibitory activity than the leaf essential oil. The MIC values of the twig oil against *S. aureus*, *E. coli*, and *C. albicans* were 4000 µg/mL, whereas the corresponding values for the leaf oil ranged from 8000 to 16,000 µg/mL. In contrast, both oils showed weaker activity against *S. enterica* and *P. aeruginosa*, with MIC values of 8000 µg/mL for the twig oil and 16,000 µg/mL for the leaf oil. The lowest IC_50_ values for the twig essential oil were recorded against the yeast *C. albicans* (2012 ± 118 µg/mL), followed by the Gram-negative bacterium *E. coli* (2193 ± 125 µg/mL) and the Gram-positive bacterium *S. aureus* (2266 ± 161 µg/mL). Much higher IC_50_ values were observed against *S. enterica* (5226 ± 264 µg/mL) and *P. aeruginosa* (4936 ± 235 µg/mL). In comparison, the leaf essential oil showed substantially weaker activity, with IC_50_ values against the five pathogenic microorganisms ranging from 5593 ± 257 to 10,593 ± 557 µg/mL (Table 2).

These results suggested that the antimicrobial efficacy of *G. lanceolata* may vary significantly depending on the plant part used for essential oil extraction. The reduced activity of the leaf oil could be associated with differences in the concentration and composition of bioactive volatile constituents responsible for antimicrobial effects. Nevertheless, the relatively high MIC values obtained for both oils indicate weaker antimicrobial potency compared with the reference compound, suggesting limited practical applicability despite the detectable inhibitory effects. Further studies are needed to identify the constituents responsible for the observed activity and to evaluate potential synergistic effects.

### 2.3. Anti-Inflammatory Activity of Glycosmis lanceolata Leaf and Twig Essential Oils

**Figure 1:**
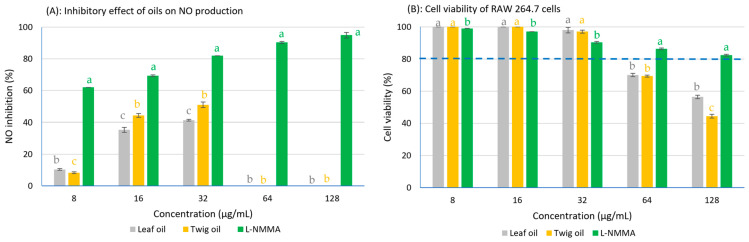
Inhibitory effect of *Glycosmis lanceolata* leaf and twig essential oils on NO production in LPS-mediated macrophage RAW 264.7 cells (**A**) and cell viability of RAW 264.7 cells (**B**) (the dashed blue line represents 80% of cell viability). Note: L-NMMA: N^G^-Methyl-L-arginine acetate; NO: nitric oxide; mean values followed by the same letter within each concentration are not statistically different at 0.05 significance level (*n* = 3). Statistical analyses were performed using the software tool IRRISTAT ver. 5.0 (International Rice Research Institute, Laguna, Philippines).

The anti-inflammatory potential of essential oils extracted from the leaves and twigs of *G. lanceolata* was evaluated using the NO assay in lipopolysaccharide (LPS)-stimulated RAW 264.7 (ATCC^®^-TIB-71TM) macrophage cells, while their cytotoxic effects were assessed using the MTT (3-(4,5-dimethylthiazol-2-yl)-2,5-diphenyl-tetrazolium bromide) assay. The RAW 264.7 cells were pre-treated with essential oils at the concentrations of 128, 64, 32, 16, and 8 μg/mL. The results demonstrated a concentration-dependent decrease in cell viability following treatment with both leaf and twig essential oils. Notably, cell viability dropped below 80% at *G. lanceolata* oil concentrations of 64 μg/mL and above, indicating considerable cytotoxicity at higher doses. Since a cell death rate exceeding 20% may lead to misleading or false-positive inhibition results, NO inhibitory activity at these concentrations was not considered reliable for anti-inflammatory evaluation. Among the tested concentrations, the strongest inhibition of NO production was observed at 32 μg/mL, with inhibition values of 41% and 51% for the leaf and twig oils, respectively. The positive control, L-NMMA, displayed a clear dose-dependent inhibitory response, with NO inhibition increasing progressively as the concentration increased. At all tested concentrations, L-NMMA exhibited significantly stronger inhibitory activity (*p* < 0.05) than both essential oil samples (Figure 1).

The essential oil extracted from the twigs of *G. lanceolata* exhibited strong inhibitory activity against NO production in LPS-stimulated RAW 264.7 macrophage cells, with an IC_50_ value of 29.7 ± 2.58 µg/mL, demonstrating considerable anti-inflammatory potential. This result indicates that the twig oil was capable of effectively suppressing NO release at relatively low concentrations while maintaining acceptable cell viability. In comparison, the positive control, N^G^-Methyl-L-arginine acetate (L-NMMA), a well-known NO synthase inhibitor, showed a significantly stronger inhibitory effect (*p* < 0.05), with an IC_50_ value of 3.76 ± 0.5 µg/mL. In contrast, the IC_50_ value of the leaf essential oil could not be determined within the tested concentration range because higher concentrations caused substantial cytotoxic effects on RAW 264.7 cells, thereby preventing accurate evaluation of its NO inhibitory activity (Table 3).

Overall, these findings suggest that the twig oil of *G. lanceolata* represents a promising natural source of anti-inflammatory compounds with potential applications in pharmaceutical and functional product development.

### 2.4. Antioxidant Activity of Glycosmis lanceolata Leaf and Twig Essential Oils

The antioxidant activity of the essential oils extracted from the leaves and twigs of *G. lanceolata* was evaluated using the 1,1-diphenyl-2-picrylhydrazyl (DPPH) free radical scavenging assay. Both oils exhibited relatively weak antioxidant activity, with the half-maximal effect concentration (EC_50_) values of the leaf and twig essential oils being 919 ± 64.4 µg/mL and 814 ± 42.5 µg/mL, respectively, indicating that there was a difference in radical scavenging capacity between the two plant parts. In contrast, the positive control quercetin displayed significantly stronger antioxidant activity (*p* < 0.05), with an EC_50_ value of 13.9 ± 0.16 µg/mL (Table 4).

The markedly higher EC_50_ values obtained for both essential oils suggest a limited ability to neutralize DPPH free radicals. This relatively weak antioxidant activity may be attributed to the chemical composition of the oils, which were dominated by terpene and terpenoid constituents. In contrast to the reference antioxidants used as positive controls, which are highly purified compounds with well-established radical-scavenging capacities, essential oils represent complex mixtures containing both active and inactive constituents. Consequently, substantially higher concentrations of the oils are required to achieve 50% DPPH inhibition. Furthermore, the major constituents identified in the present study, including neral, geranial, and (E)-β-caryophyllene, generally exhibit lower hydrogen-donating and electron-transfer capacities than phenolic antioxidants such as Trolox, quercetin, or ascorbic acid. Therefore, the relatively high EC_50_ values observed in this study are not unexpected and should be interpreted in the context of the chemical nature of essential oils rather than as a direct indication of poor biological activity. It should also be noted that the DPPH assay primarily reflects the capacity of compounds to donate hydrogen atoms or electrons in a homogeneous reaction system and may underestimate the antioxidant potential of non-phenolic terpenoids. Therefore, the antioxidant capacity of the essential oils may not be fully represented by the DPPH assay alone.

## 3. Discussion

The essential oil yields obtained from the leaves and twigs of *G. lanceolata* collected in Son La Province in the present study (0.201% and 0.064%, respectively) were lower than those previously reported for leaf samples collected in Da Nang, Vietnam (0.35%) by Vo et al. [28]. Nevertheless, the higher essential oil yield observed in leaves compared with twigs is consistent with previous findings for *G. mauritiana* reported by Hoang et al. [20]. Variations in essential oil yield may be influenced by multiple factors, including geographical origin, climatic conditions, harvesting season, plant developmental stage, and extraction methodology.

The chemical compositions of the leaf and twig essential oils of *G. lanceolata* showed qualitative similarities but considerable quantitative differences in the relative abundance of major constituents and chemical groups. Both oils contained characteristic compounds such as linalool (0.5% and 7.9%), methyleugenol (2.5% and 9.3%), (*E*)-*β*-caryophyllene (10.2% and 5.7%), *β*-bisabolene (23.7% and 11.6%), spathulenol (3.3% and 3.5%), caryophyllene oxide (3.0% and 6.6%), and brevifolin (21.3% and 12.7%) in the leaf and twig oils, respectively. However, the twig oil was characterized by markedly higher proportions of oxygenated compounds and benzenoids, whereas the leaf oil was dominated by sesquiterpene hydrocarbons. Such differences in chemical profiles are likely to contribute significantly to variations in aroma characteristics as well as biological activities between the two oils.

Previous studies on *G. lanceolata* from Da Nang, Vietnam [28] reported a substantially different chemical profile for the leaf essential oil, consisting mainly of *β*-myrcene (6.5%), limonene (59.3%), *β*-caryophyllene (8.8%), and bicyclogermacrene (7.6%). The substantial differences in both essential oil yield and chemical composition between the present study and the report of Vo et al. [28] may reflect the influence of environmental and ecological factors. Secondary metabolite biosynthesis in aromatic plants is highly responsive to altitude, temperature, rainfall patterns, soil characteristics, and light conditions. Son La Province is located in the mountainous northwestern region of Vietnam, whereas Da Nang is situated in a coastal tropical area with markedly different climatic conditions. Such environmental differences may alter the activity of terpene biosynthetic pathways and consequently affect both the yield and composition of essential oils. However, direct comparison with the present study should be made cautiously because retention index (RI) values for the identified compounds were not provided in that report, making confirmation of compound identification less reliable.

The genus *Glycosmis* is known to exhibit remarkable chemical diversity in essential oil composition depending on species, plant organ, geographical origin, and environmental conditions. For example, the leaf essential oil of *G. lucida* collected in China was reported to contain elixene (19.81%), anethole (12.05%), verbenone (10.32%), and spathulenol (10.68%) as major constituents [18]. In Vietnam, *G. mauritiana* was characterized by high levels of myristicin in both leaves (21.28%) and twig (17.25%), together with (Z)-13-docosenamide accounting for 9.07% in leaves and 13.41% in branches [20]. Similarly, the aerial parts of *G. parviflora* collected in China were dominated by (*Z*)-caryophyllene (20.6%) and methyl isoeugenol (11.1%) [19]. Among the species of the genus, *G. pentaphylla* has been the most extensively investigated and has shown substantial variation in essential oil composition across different plant parts and geographical regions. In India, bark oil was rich in 2-undecanone (58.1%) and 2-tridecanone (23.4%), whereas leaf oil contained mainly 2-tridecanone (36.8%), 6,10,14-trimethyl-2-pentadecanone (13.1%), and hexadecanoic acid (25.6%). In contrast, seed oil was characterized by high concentrations of linalool (24.7%) and terpinen-4-ol (19.2%) [15]. Additional studies from India reported markedly different compositions in leaf oils, including benzaldehyde oxime (15.66%) and bicyclo[6.1.0]non-1-ene (18.93%) [29] (however, these two compounds are unlikely in an essential oil), or phytol (28.03%), bicyclo[5.2.0]nonane, 2-methylene-4,8,8-trimethyl-4-vinyl- (10.93%) (however, this compound is unlikely in an essential oil), and 1,19-eicosadiene (9.84%) [16]. In Vietnam, the leaf essential oil of *G. pentaphylla* was reported to contain high levels of *β*-ocimene (23.10%) and caryophyllene (16.14%) [21] (however, the elution orders are not correct in that literature), reflecting the predominance of monoterpenes and sesquiterpenes commonly observed within the genus. Furthermore, *G. puberula* var. *eberhardtii* from Vietnam was found to possess linalool (12.2%), %), (*E*)-*β*-caryophyllene (25.1%), and α-humulene (28.3%) as the major constituents in leaf essential oil [22], highlighting the occurrence of valuable aromatic monoterpenes with potential applications in perfumery and cosmetic industries (Table 5).

Essential oils from the genus *Glycosmis* in general and from *G. lanceolata* species in particular exhibit considerable chemical diversity among species and even within the same species, depending on geographical origin, plant part, and ecological conditions. These oils are mainly composed of monoterpenes, sesquiterpenes, benzenoid derivatives, and ketone compounds, many of which are species-specific. However, it should be noted specifically that many of the previous works are unreliable, as mentioned above. The variation in the chemical composition of species reflects the biochemical plasticity of the genus and is important for chemotaxonomic studies, as well as for identifying valuable bioactive compounds with potential applications in pharmaceuticals, food preservation, perfumery, and cosmetics. In this study, the differences in antimicrobial, anti-inflammatory, and antioxidant activity between the leaf and twig essential oils of *G. lanceolata* can be explained primarily by the differences in chemical composition and relative content of the major compounds in the two essential oils.

Numerous studies have shown that essential oils inhibit microbial growth through various mechanisms, including disruption of cell membranes, increased membrane permeability, and interference with microbial metabolic processes [30]. Due to their complex composition of bioactive volatile compounds, essential oils may also reduce the risk of microbial resistance compared with conventional antibiotics. Therefore, evaluating the antimicrobial activity of essential oils against pathogenic microorganisms is important for identifying natural agents with potential applications in food preservation, pharmaceuticals, cosmetics, and other eco-friendly products. Such studies contribute valuable scientific evidence for the development of sustainable natural products beneficial to human health and daily life.

Inflammation is a complex protective response of the host microcirculatory system to tissue injury caused by a wide range of abiotic and biotic factors, including physical damage, chemical irritants, and microbial invasion. Although acute inflammation is essential for maintaining tissue homeostasis and promoting healing, prolonged or dysregulated inflammatory responses may contribute to the development of numerous chronic disorders, such as cardiovascular diseases, autoimmune diseases, neurodegenerative disorders, and cancer [31]. Among the immune cells involved in inflammatory processes, macrophages play a pivotal role by producing a variety of pro-inflammatory mediators and cytokines. One of the major inflammatory mediators is nitric oxide (NO), which is generated in large amounts by inducible nitric oxide synthase (iNOS) during inflammatory stimulation. Excessive NO production has been associated with oxidative stress, tissue injury, and the progression of severe pathological conditions, including rheumatoid arthritis, carcinogenesis, and neurodegenerative diseases [32,33,34]. Therefore, the discovery of novel inhibitors of NO production is of significant interest. In recent years, essential oils have emerged as promising sources of bioactive compounds due to their diverse pharmacological properties, including anti-inflammatory, antioxidant, and antimicrobial activities.

The twig essential oil exhibited markedly stronger biological activities than the leaf essential oil, particularly in terms of antimicrobial and anti-inflammatory effects. This enhanced bioactivity is likely associated with the substantially higher abundance of oxygenated monoterpenes and oxygenated sesquiterpenes in the twig oil. According to the chemical composition analysis, the twig essential oil contained significantly greater proportions of oxygenated monoterpenes (8.4%) and oxygenated sesquiterpenes (23.5%) compared with the leaf essential oil (0.8% and 12.7%, respectively). Previous studies have demonstrated that oxygenated terpene derivatives generally possess stronger biological activities than terpene hydrocarbons because the presence of oxygen-containing functional groups enhances their interactions with microbial cell membranes and inflammatory enzymes [35,36]. Among the major constituents, linalool was particularly prominent in the twig essential oil (7.9%), whereas it occurred only in trace amounts in the leaf oil (0.5%). Linalool is a well-known monoterpene alcohol with diverse biological properties, including antimicrobial, anti-inflammatory, and antioxidant activities. It has been reported to disrupt microbial membrane integrity, increase membrane permeability, and induce leakage of intracellular constituents, thereby inhibiting microbial growth [37,38]. In addition, linalool has been shown to suppress nitric oxide (NO) production and reduce the expression of pro-inflammatory cytokines through modulation of the NF-κB and iNOS signaling pathways in activated macrophages [39]. Therefore, the relatively high concentration of linalool in the twig essential oil may play a key role in its superior antimicrobial and anti-inflammatory activities. Furthermore, the enhanced biological activity of the twig oil may not depend solely on individual major compounds but also on synergistic interactions among volatile constituents. Essential oils are complex mixtures in which both major and minor components can interact synergistically, additively, or antagonistically, thereby influencing overall bioactivity. Several studies have emphasized that the antimicrobial and anti-inflammatory properties of essential oils are often the result of combined effects among multiple constituents rather than the activity of a single dominant compound [40]. Such synergistic interactions may contribute significantly to the stronger biological activities observed in the twig essential oil of *G. lanceolata*.

To the best of our knowledge, studies investigating the antimicrobial and NO-inhibitory activity of essential oils derived from *Glycosmis* species remain extremely limited. Previous research on this genus has primarily focused on crude extracts and isolated carbazole alkaloids, many of which have demonstrated notable antimicrobial activity and anti-inflammatory activity through the suppression of NO production in activated macrophages [14]. These findings suggest that *Glycosmis* species represent a valuable source of bioactive secondary metabolites with potential therapeutic applications. In contrast to earlier studies centered mainly on non-volatile extracts and purified compounds, the present study provides novel evidence regarding the anti-inflammatory potential of essential oils obtained from the leaves and twigs of *G. lanceolata*. The observed NO inhibitory activity, particularly that of the twig essential oil, highlights the potential contribution of volatile constituents to the biological properties of this species. Although the activity of the essential oils was lower than that reported for some isolated carbazole alkaloids, the results remain significant given the complex and naturally occurring composition of essential oils. Furthermore, the differential activities observed between leaf and twig oils suggest that variations in chemical composition may strongly influence their pharmacological effects. Overall, these findings expand the current understanding of the biological activities of *G. lanceolata* and support its potential as a promising natural source of anti-inflammatory agents for future pharmaceutical, nutraceutical, and functional product development.

The antioxidant activity of essential oils has attracted considerable scientific and industrial interest due to their potential applications in food preservation, pharmaceutical formulations, and health promotion. Essential oils may help protect foods against oxidative deterioration by inhibiting lipid peroxidation and reducing the toxic effects of reactive oxidant species, thereby improving product stability and shelf life [41]. In addition to their technological applications in food systems, essential oils have also been recognized as promising natural sources of bioactive compounds capable of scavenging free radicals and reducing oxidative stress in biological systems. Oxidative stress results from an imbalance between the production of reactive oxygen species (ROS) and the antioxidant defense mechanisms of the body. Excessive accumulation of free radicals can damage essential biomolecules such as lipids, proteins, and nucleic acids, ultimately leading to cellular dysfunction and tissue injury. Increasing evidence suggests that oxidative stress plays a crucial role in the pathogenesis of numerous chronic and degenerative diseases, including neurodegenerative disorders, cancer, cardiovascular diseases, diabetes, premature aging, and immune system decline [42,43]. Consequently, natural antioxidants capable of neutralizing free radicals have become important targets in the search for safer and more effective therapeutic and preventive agents.

The weak antioxidant activity observed in the essential oils may be attributed to their chemical composition, which is dominated primarily by terpene and terpenoid constituents. Although certain terpenes have been reported to possess antioxidant properties, their activity is generally lower than those of phenolic compounds because they lack hydroxyl groups capable of efficiently donating hydrogen atoms or electrons to stabilize free radicals [36,44]. In contrast, quercetin, a flavonoid rich in phenolic hydroxyl groups, is recognized as a highly potent antioxidant due to its strong free radical scavenging and metal-chelating abilities [45]. Therefore, the significant difference between the antioxidant activities of quercetin and *G. lanceolata* essential oils is consistent with the known structure–activity relationships of natural antioxidants. Similar observations have been reported for many plant essential oils rich in monoterpenes and sesquiterpenes, where moderate or weak antioxidant activity was associated with the low abundance of phenolic constituents [36,40]. On the other hand, the presence of a high concentration of β-bisabolene in the essential oil may also be considered one of the reasons for its weak antioxidant activity [46]. Despite their limited direct radical scavenging capacity, essential oils may still contribute beneficial biological effects through synergistic interactions among their volatile constituents or in combination with other natural antioxidants. Moreover, antioxidant activity in biological systems may involve multiple mechanisms beyond DPPH radical scavenging, including modulation of oxidative enzymes, inhibition of lipid peroxidation, and enhancement of endogenous antioxidant defenses.

Though both essential oils of *G. lanceolata* exhibited relatively weak antioxidant activity in the DPPH radical scavenging assay, the twig essential oil demonstrated slightly greater activity than the leaf oil. This difference may be attributed to the higher abundance of oxygenated compounds in the twig oil, particularly linalool (7.9% vs. 0.5%), methyleugenol (9.3% vs. 2.3%), and caryophyllene oxide (6.6% vs. 3.0%). These oxygenated constituents are known to possess stronger radical scavenging potential because they can more readily donate hydrogen atoms or electrons to stabilize free radicals compared with non-oxygenated terpene hydrocarbons [36]. In contrast, the leaf essential oil was characterized by higher levels of sesquiterpene hydrocarbons, including *β*-bisabolene (23.7% vs. 11.6%), brevifolin (21.3% vs. 12.7%), and (*E*)-*β*-caryophyllene (10.2% vs. 5.7%). Although sesquiterpene hydrocarbons may contribute to certain biological activities, they generally exhibit lower antioxidant and antimicrobial potency than oxygenated terpenes due to the absence of functional groups capable of efficient hydrogen or electron donation [44]. Consequently, despite its relatively high sesquiterpene hydrocarbon content, the leaf essential oil displayed weaker overall biological activity than the twig essential oil.

Research on the general biological properties, particularly antioxidant capacity, of essential oils from species within the genus *Glycosmis* remains relatively limited. A previous study showed that essential oil from the leaves of *G. pentaphylla* demonstrated notably stronger antioxidant activity (IC_50_ = 21.92 µg/mL) compared to that of *G. lanceolata* reported in the present study [17]. This may be attributed to marked differences in chemical composition. Antioxidant activity is strongly influenced not only by the abundance of oxygenated terpenoids but also by the presence of phenolic constituents and other compounds capable of donating hydrogen atoms or electrons. Therefore, interspecific differences in biosynthetic pathways and secondary metabolite profiles within the genus *Glycosmis* may account for the considerable variation in antioxidant capacity observed among species. Several studies have reported significant insect repellent and larvicidal properties [18,19,29], and herbicidal activity [22] of essential oils from other *Glycosmis* species.

These results are generally consistent with the growing body of evidence indicating that members of the genus *Glycosmis* possess bioactive potentials. However, the magnitude of activity varies considerably among species and extracts. Such variability is likely attributable to differences in phytochemical composition, extraction methods, and the relative abundance of specific bioactive metabolites. Overall, the present findings suggest that the essential oils of *G. lanceolata* are not potent natural antioxidants when evaluated by the DPPH assay alone. Nevertheless, they may still possess complementary or synergistic biological functions that could support their potential applications in pharmaceutical, cosmetic, or functional product development. Although the present study provides initial evidence of the antioxidant, anti-inflammatory, and antimicrobial potential of *G. lanceolata* essential oils, further investigations employing additional antioxidant assays, mechanistic anti-inflammatory studies, detailed antimicrobial evaluations (including MIC and MBC/MFC determinations), and in-depth safety assessments are warranted to obtain a more comprehensive understanding of their biological activities and potential applications.

## 4. Materials and Methods

### 4.1. Plant Source

Within a research program evaluating medicinal plants under the forest canopy in northern Vietnam, we investigated and evaluated the chemical composition and antimicrobial, antioxidant, and anti-inflammatory activities of essential oils from *Glycosmis lanceolata* (Blume) D. Dietr. belonging to the Rutaceae family. The samples of fresh leaves and twigs of this species were collected in Son La province in June 2024. Voucher specimen (SL04-19) was identified by Prof. Dr. Tran The Bach and deposited at the Herbarium of the Institute of Biology (HN), Vietnam Academy of Science and Technology.

### 4.2. Essential Oil Isolation

Fresh leaves (2.38 kg) and twigs (1.34 kg) of *G. lanceolata* were shredded and subjected to hydrodistillation for 3.5 h using a Clevenger-type apparatus [47]. For each batch of distillation, the plant material was immersed in 1.2 L of distilled water and heated to boiling under atmospheric pressure. During hydrodistillation, tap water was continuously circulated through the glass condenser to cool the vapor stream, thereby condensing the volatilized essential oil constituents and water into a liquid distillate for subsequent separation. The distillations were performed in triplicate for leaf samples and in duplicate for twig samples. The obtained essential oil was then separated from the aqueous phase, transferred into amber glass vials, and stored at −5 °C for subsequent analysis.

### 4.3. Essential Oil GC-MS and GC-FID Analysis

Essential oil samples were analyzed using GC/MS-FID on an Agilent 7890A gas chromatograph coupled to a 5975C Mass Selective Detector (Agilent Technologies, Santa Clara, CA, USA). Chromatographic separation was achieved on an HP-5MS fused silica capillary column (60 m × 0.25 mm internal diameter, 0.25 μm film thickness; Agilent Technologies, Santa Clara, CA, USA). Helium served as the carrier gas at a constant flow rate of 1.0 mL/min. The injector temperature was maintained at 250 °C, and 1 μL of sample was injected in split mode with a ratio of 1:100. The oven temperature was initially set at 60 °C and programmed to increase to 260 °C at a rate of 4 °C/min. The detector temperature was kept at 230 °C. For mass spectrometric analysis, the interface temperature was 280 °C, and ionization was performed by electron ionization (EI) at 70 eV, with a scan speed of 4.0 scans/s over a mass range of 35–450 Da. The GC–MS analysis was conducted in triplicate for each essential oil sample. FID measurements were carried out under the same chromatographic conditions, with the detector temperature maintained at 250 °C. Compound identification was based on comparison of relative retention indices (calculated by co-injection with a homologous series of n-alkanes, C7–C30) and mass spectral fragmentation patterns with those reported in standard libraries (NIST08, Wiley09, and HPCH1607) [48,49,50]. Data processing was performed using MassFinder 4.0 software. Relative percentages of the components were determined from FID peak areas without applying correction factors.

### 4.4. Tested Microbial Strains

A total of seven microbial strains were selected for antimicrobial screening, including three Gram-positive bacteria (*Staphylococcus aureus* ATCC 13709, *Bacillus subtilis* ATCC 6633, and *Lactobacillus fermentum* VTCC N4), three Gram-negative bacteria (*Salmonella enterica* VTCC, *Escherichia coli* ATCC 25922, and *Pseudomonas aeruginosa* ATCC 15442), and the yeast (*Candida albicans* ATCC 10231). ATCC strains were sourced from the American Type Culture Collection (Manassas, VA, USA), while VTCC strains were supplied by the Vietnam Type Culture Collection, Institute of Microbiology and Biotechnology, Vietnam National University, Hanoi.

### 4.5. Screening of Antimicrobial Activity of Essential Oil

The antimicrobial activity of the essential oils was assessed by determining the minimum inhibitory concentration (MIC) and the concentration required to inhibit 50% of microbial growth half-maximal inhibitory concentration (IC_50_) using the broth microdilution assay performed in triplicate [51,52]. Essential oils were prepared in dimethyl sulfoxide (DMSO) and serially diluted with sterile distilled water to obtain five concentrations: 16,000, 4000, 1000, 250, and 62.5 μg/mL. Aliquots of each dilution were dispensed into 96-well microplates. Bacterial inocula were prepared in double-strength Mueller–Hinton broth or double-strength tryptic soy broth, whereas fungal cultures were maintained in double-strength Sabouraud dextrose broth. Cell densities were standardized to 5 × 10^5^ CFU/mL for bacteria and 1 × 10^3^ CFU/mL for fungi prior to testing. Wells containing only culture medium (without essential oil dilutions and microorganisms) served as negative controls, while wells containing microbial suspensions without essential oils were used as positive controls.

Following incubation at 37 °C for 24 h, MIC values were recorded as the lowest concentrations at which no visible microbial growth was observed. Growth inhibition was quantified from optical density measurements obtained with an EPOCH2C spectrophotometer (BioTek Instruments, Winooski, VT, USA). IC_50_ values were subsequently estimated from the inhibition percentages using Raw Data software (Intercity Business Park Mechelen Noord, Mechelen, Belgium) according to the following equations:

(1) $$\%\;inhibition=\frac{{OD}_{control\left(+\right)}-\;{OD}_{test\;agent}}{{OD}_{control\left(+\right)}-{OD}_{control\left(-\right)}}\times 100\%$$

(2) $${IC}_{50}={High}_{Conc\;}-\frac{{(High}_{Inh\%\;}-50\%)\;{(High}_{Conc}-{Low}_{Conc})}{\left({High}_{Inh\%}-{Low}_{Inh\%}\right)}$$

where *OD_control_*_(+)_ is the optical density representing the absorbance of the positive control, consisting of microbial cells grown in the absence of the tested essential oil or antimicrobial agent, *OD_test agent_* is the optical density corresponding to the absorbance recorded for wells containing microorganisms exposed to a defined concentration of the test samples (antimicrobial agent or essential oil), and *OD_control_*_(−)_ is the optical density denoting the absorbance of the negative control containing only culture medium (without cells). For IC_50_ calculations, *High_Conc_*/*Low_Conc_* are the high and low concentrations referring to the two concentrations of the test agent or essential oil immediately above and below 50% inhibition, respectively, while *High_Inh_*_%_/*Low_Inh_*_%_ are the percentages of microbial growth inhibition at high and low concentrations, respectively.

To validate the assay, standard antimicrobial agents were included as reference compounds: Ampicillin was employed against Gram (+) bacteria, yielding IC_50_ and MIC values ranging from 0.02 to 3.62 µg/mL, and 0.125–32.0 µg/mL, respectively. Cefotaxime was used for Gram (–) bacteria, with corresponding IC_50_ and MIC values of 0.007–4.34 µg/mL and 0.5–32.0 µg/mL. Antifungal activity was benchmarked using nystatin, which exhibited an average IC_50_ of 1.32 µg/mL and an MIC of 8.0 µg/mL.

### 4.6. Assay for NO Inhibitory Effect of Essential Oil Using RAW264.7 Cells

The murine macrophage cell line RAW 264.7 (ATCC^®^-TIB-71TM) were maintained in Dulbecco’s Modified Eagle Medium (DMEM) supplemented with 10% fetal bovine serum (FBS), 100 U/mL penicillin, 100 μg/mL streptomycin, and 0.25 μg/mL Gibco (Grand Island, NY, USA) amphotericin B. Cells were dispensed into 96-well microplates at a density of 2 × 10^5^ cells/well and incubated for 24 h under a humidified atmosphere containing 5% carbon dioxide (CO_2_) at 37 °C. The growth medium was subsequently replaced with serum-free DMEM (DMEM without FBS), followed by a further 3 h equilibration period.

To assess anti-inflammatory activity, the cells were exposed to essential oil samples or the reference inhibitor (positive control) N^G^-methyl-L-arginine acetate (L-NMMA) at concentrations of 128, 64, 32, 16, and 8 μg/mL (corresponding to 2^7^, 2^6^, 2^5^, 2^4^, and 2^3^ µg/mL) for 2 h. The cells were then stimulated with 10 μg/mL lipopolysaccharides (LPS) and incubated for an additional 24 h prior to stimulation. Inflammatory responses were induced by treatment with lipopolysaccharide (LPS, 10 μg/mL), and the cultures were incubated for an additional 24 h. Nitric oxide (NO) production was determined using the Griess Reagent System (Promega Corporation, Fitchburg, WI, USA), with L-NMMA (Sigma-Aldrich, St. Louis, MO, USA) employed as the reference standard (positive control). The nitrite (NO_2_^−^) accumulation in the culture supernatant was quantified spectrophotometrically at 540 nm (absorbance—A540) using a microplate reader, and calculated from a sodium nitrite (NaNO_2_) calibration curve.

All measurements were performed in triplicate. The half-maximal inhibitory concentration (IC_50_) values for inhibition of NO production were estimated using TableCurve 2Dv4 software. In parallel, cell viability was examined using the MTT colorimetric assay (3-(4,5-dimethylthiazol-2-yl)-2,5-diphenyl-tetrazolium bromide) [53,54] to exclude false-positive effects resulting from cytotoxicity.

The formulas used to calculate the percentage of cell viability (3) and percentage of NO production inhibition (4) are as follows:

(3) $$Cell\;viability\;\left(\%\right)=100\%-\frac{{OD}_{control(+)}-\;{OD}_{test\;agent}}{{OD}_{control(+)}-{OD}_{control(-)}}\;\times \;100\%$$

(4) $$NO\;production\;inhibition\;\left(\%\right)=\frac{{OD}_{control(+)}-{OD}_{test\;agent}}{{OD}_{control(+)}-{OD}_{control(-)}}\times 100\%$$

where OD_control(+)_ is the optical density of positive control sample (cells in medium without cytotoxic agent/essential oil), OD_test agent_ is the optical density of test samples (each sample corresponds to a known concentration of cytotoxic agent/essential oil), and OD_control(−)_ is the optical density of negative control sample (culture medium without cells).

The percentage of NO production inhibition will be calculated for the sample at concentrations where cell viability is greater than 80%, to avoid obtaining false positive results.

### 4.7. Assay for Antioxidant Activity of Essential Oil

The antioxidant activity of the tested samples was evaluated using the 1,1-diphenyl-2-picrylhydrazyl (DPPH) free radical scavenging assay. The reduction in DPPH radicals, indicated by a decrease in absorbance, was measured spectrophotometrically. A 1 mM DPPH solution was prepared in methanol (MeOH), while the test samples were diluted in deionized water. Serial concentrations of the test samples (16,000, 4000, 1000, 250, and 62.5 μg/mL), and of the Quercetin (Sigma) reference material (128, 32, 8, and 2 μg/mL) were prepared in a 96-well microplate and mixed with the DPPH solution. The reaction mixtures were incubated at 37 °C for 30 min. After incubation, absorbance was measured at 517 nm using a BioTek spectrophotometer (BioTek Instruments, Inc., Winooski, VT, USA).

The percentage of DPPH radical scavenging capacity (% SC) was calculated using the following equation:

Scavenging capacity (%) = (OD_control_ − OD_test agent_)/OD_control_ (5)

where OD_control_: Optical density of control sample (medium without antioxidant agent/essential oil); OD_test agent_: Optical density of test samples (each sample corresponds to a known concentration of antioxidant agent/essential oil).

The half-maximal effect concentration (EC_50_) value, defined as the concentration of the tested sample required to achieve 50% scavenging capacity, was determined by linear regression analysis in Excel using the percentage scavenging activity against sample concentrations [55,56]. All experiments were performed in triplicate (*n* = 3).

### 4.8. Chemicals and Reagents

All chemicals and reagents used in this study were of analytical or chromatographic grade. Helium (99.999% purity, VSG Gas, Ho chi Minh city, Vietnam) was used as the carrier gas for GC–MS and GC–FID analyses, while *n*-Hexan (Sigma-Aldrich, St. Louis, MO, USA) was used to dilute essential oils. A homologous series of *n*-alkanes (C7–C30) was employed for the calculation of retention indices (RI). Dimethyl sulfoxide (DMSO) was used for the preparation of essential oil solutions in antimicrobial and anti-inflammatory assays. For antimicrobial evaluation, Mueller–Hinton broth (MHB), tryptic soy broth (TSB), and Sabouraud dextrose broth (SDB) were used as culture media. Ampicillin, cefotaxime, and nystatin served as positive controls for Gram-positive bacteria, Gram-negative bacteria, and fungi, respectively. For the anti-inflammatory assay, Dulbecco’s Modified Eagle Medium (DMEM), fetal bovine serum (FBS), penicillin–streptomycin solution, amphotericin B, lipopolysaccharide (LPS), NG-methyl-L-arginine acetate (L-NMMA), Griess reagent, sodium nitrite (NaNO_2_), and 3-(4,5-dimethylthiazol-2-yl)-2,5-diphenyl-tetrazolium bromide (MTT) were employed. RAW 264.7 murine macrophage cells were obtained from the American Type Culture Collection (ATCC). For antioxidant evaluation, 2,2-diphenyl-1-picrylhydrazyl (DPPH), methanol (MeOH), quercetin, and deionized water were used. Unless otherwise stated, all chemicals and reagents were purchased from Sigma-Aldrich (St. Louis, MO, USA) and Merck (Darmstadt, Germany).

### 4.9. Statistical Analysis

Data of *G. lanceolata* were subjected to one-way analysis of variance (ANOVA) to compare with the standard reference materials. Significant differences were further evaluated by comparing means using the least significant difference (LSD) test at *p* ≤ 0.05. Statistical analyses were performed using IRRISTAT version 5.0 (International Rice Research Institute, Philippines).

## 5. Conclusions

Essential oils obtained from fresh leaves and twigs of *G. lanceolata* collected in Son La province (Vietnam) exhibited broadly similar chemical profiles, differing primarily in the relative abundance of individual constituents. The dominant compounds included β-bisabolene, brevifolin, and/or (*E*)-β-caryophyllene, although their proportions varied between plant parts. Specifically, leaf oil was characterized by a higher content of sesquiterpene hydrocarbons, whereas twig oil contained greater amounts of oxygenated compounds and benzenoid derivatives. These compositional differences appear to underlie the observed variation in biological activities. Neither essential oil demonstrated inhibitory effects against the beneficial microorganisms *B. subtilis* and *L. fermentum* within the tested concentration range. In contrast, both oils exhibited weak activity against five pathogenic strains—*S. aureus*, *S. enterica*, *E. coli*, *P. aeruginosa*, and *C. albicans*—with the twig essential oil showing consistently stronger antimicrobial effects, as evidenced by lower IC_50_ and MIC values compared to the leaf oil. A similar trend was observed for anti-inflammatory and antioxidant activities, where the twig oil demonstrated greater inhibition of NO production and stronger DPPH radical scavenging capacity. These findings suggest that oxygenated terpenoids and benzenoid compounds may play a more prominent role in determining the biological efficacy of *G. lanceolata* essential oils than hydrocarbon sesquiterpenes. Overall, this study contributes valuable new data to the still-limited phytochemical and bioactivity database of *G. lanceolata*. The results highlight the potential of this species as a promising source of bioactive compounds for applications in pharmaceuticals, cosmetics, and functional foods, while also providing a foundation for future studies focusing on mechanism elucidation, compound isolation, and product development. It should also be noted that the biological activities were evaluated using in vitro assays only. Although these methods provide valuable preliminary information regarding the pharmacological potential of the essential oils, they do not fully reflect the complexity of biological responses in vivo. Furthermore, the specific constituents responsible for the observed activities were not individually evaluated. Future studies involving bioactivity-guided fractionation, mechanistic investigations, and in vivo models would be valuable for identifying the active constituents and clarifying their modes of action. Specifically, mechanistic analyses of inflammation-related pathways, including NF-κB, iNOS, and COX-2 signaling, are required to confirm the compounds responsible for the observed effects and are necessary to confirm the pharmacological relevance and practical applicability of these essential oils.

## Figures and Tables

**Table 1:** Chemical composition (%) of *Glycosmis lanceolata* leaf and twig essential oils.

Compounds ^a^	RI ^b^	Leaf Oil ^c^	Twig Oil ^c^
*α*-Pinene	939	0.4	1.0
Myrcene	992	Tr	0.2
Limonene	1034	Tr	0.2
*β*-Phellandrene	1035	0.1	0.2
(*E*)-*β*-Ocimene	1048	0.3	0.7
Linalool	1102	0.5	7.9
(*E*)-4,8-Dimethylnona-1,3,7-triene	1117	0.1	0.2
Terpinen-4-ol	1185	Tr	0.3
*α*-Terpineol	1197	Tr	0.2
*α*-Cubebene	1360	0.2	Tr
*α*-Copaene	1389	0.8	0.4
*cis*-*β*-Elemene	1403	2.2	0.9
Methyleugenol	1408	2.5	9.3
Methyl *N*-methylanthranilate	1420	Tr	5.3
(*E*)-*β*-Caryophyllene	1437	10.2	5.7
*trans*-*α*-Bergamotene	1445	0.3	Tr
Geranylacetone	1456	0.3	Tr
(*Z*)-*β*-Farnesene	1460	1.0	0.4
(*E*)-*β*-Farnesene	1465	0.3	Tr
*α*-Humulene	1471	1.8	1.2
*γ*-Muurolene	1490	0.3	0.3
Germacrene D	1498	3.9	0.3
(*E*)-Methyl isoeugenol	1501	Tr	0.7
*trans*-Muurola-4(14),5-diene	1510	0.3	Tr
4-*epi*-Cubebol	1510	Tr	0.2
Viridiflorene	1511	Tr	0.2
*α*-Muurolene	1513	Tr	0.3
Bicyclogermacrene	1514	2.2	Tr
*β*-Bisabolene	1518	23.7	11.6
*γ*-Cadinene	1531	0.5	0.2
*β*-Sesquiphellandrene	1534	1.5	0.6
δ-Cadinene	1537	0.3	0.4
*cis*-Calamenene	1538	Tr	0.2
Elemicin	1560	1.5	2.5
Elemol	1563	0.4	0.5
(*E*)-Nerolidol	1569	0.4	0.6
Dendrolasin	1583	0.2	Tr
Mintoxide	1587	Tr	0.2
Germacrene D-4-ol	1594	0.2	Tr
Spathulenol	1597	3.3	3.5
Caryophyllene oxide	1605	3.0	6.6
Zingiberenol	1623	0.4	Tr
Copaborneol	1625	Tr	0.4
Cedrol	1626	Tr	1.0
Humulene epoxide II	1631	0.5	1.0
(3*Z*)-Hexenyl phenyl acetate	1641	0.2	Tr
1-*epi*-Cubenol	1646	Tr	1.0
*γ*-Eudesmol	1650	Tr	0.4
Caryophylla-3(15),7(14)-dien-6-ol	1653	Tr	0.5
*epi*-*α*-Cadinol (=τ-Cadinol)	1658	2.3	3.6
*α*-Muurolol (=δ-Cadinol)	1663	0.4	0.8
*α*-Cadinol	1672	0.7	1.6
*neo*-Intermedeol	1675	0.2	0.3
Intermedeol	1682	0.5	0.9
Brevifolin	1689	21.3	12.7
Eudesma-4(15),7-dien-1β-ol	1706	Tr	0.4
(*E,E*)-Farnesol	1727	0.2	Tr
6,10,14-Trimethylpentadecan-2-one	1847	0.5	Tr
Phytol	2117	1.2	0.4
Total		91.1	88.0
Monoterpene hydrocarbons		0.8	2.3
Oxygenated monoterpenes		0.8	8.4
Sesquiterpene hydrocarbons		49.5	22.5
Oxygenated sesquiterpenes		12.7	23.5
Oxygenated diterpenes		1.2	0.4
Benzenoids		25.5	30.7
Others		0.6	0.2
Number of identified compounds		42	43

Note: ^a^ elution order of compounds on HP-5MS column; ^b^ RI: retention index of compounds on the HP-5MS column; ^c^ standard deviations were insignificant and excluded from the table to avoid congestion (*n* = 3); Tr: trace (concentration < 0.1%).

**Table 2:** MIC and IC_50_ values of *Glycosmis lanceolata* leaf and twig essential oils against tested microorganisms.

Samples	Parameters	The Concentration of Essential Oil Inhibiting the Tested Microorganisms (µg/mL)						
		Gram (+) Bacteria			Gram (−) Bacteria			Yeast
		*Staphylococcus aureus*	*Bacillus subtilis*	*Lactobacillus fermentum*	*Salmonella enterica*	*Escherichia coli*	*Pseudomonas aeruginosa*	*Candida albicans*
Leaf oil	IC_50_	6630 ± 246	>16,000	>16,000	9628 ± 461	5593 ± 257	10,593 ± 557	10,000 ± 515
	MIC	8000 ± 0.0	>16,000	>16,000	16,000 ± 0.0	8000 ± 0.0	16,000 ± 0.0	16,000 ± 0.0
Twig oil	IC_50_	2266 ± 161	>16,000	>16,000	5226 ± 264	2193 ± 125	4936 ± 235	2012 ± 118
	MIC	4000 ± 0.0	>16,000	>16,000	8000 ± 0.0	4000 ± 0.0	8000 ± 0.0	4000 ± 0.0
Ampicillin	IC_50_	0.02 ± 0.005	3.62 ± 0.15	1.03 ± 0.07				
	MIC	0.125 ± 0.0	32 ± 0.0	32 ± 0.0				
Cefotaxime	IC_50_				0.43 ± 0.05	0.007 ± 0.002	4.34 ± 0.15	
	MIC				32 ± 0.0	0.5 ± 0.0	8 ± 0.0	
Nystatin	IC_50_							1.32 ± 0.05
	MIC							8 ± 0.0

**Table 3:** IC_50_ values for inhibition of nitric oxide (NO) production by *Glycosmis lanceolata* leaf and twig essential oils in LPS-stimulated RAW 264.7 macrophages.

Inhibition of NO Production	Leaf Oil	Twig Oil	L-NMMA *
IC_50_ (µg/mL)	UD	29.7 ± 2.58 ^a^	3.76 ± 0.5 ^b^

Note: * N^G^-Methyl-L-arginine acetate (reference material); UD = undetermined. Mean values followed by different letters within the table are statistically different at the 0.05 significance level (*n* = 3). Statistical analysis was performed using the software tool IRRISTAT ver. 5.0 (International Rice Research Institute, Laguna, Philippines).

**Table 4:** EC_50_ values for DPPH radical scavenging activity of *Glycosmis lanceolata* leaf and twig essential oils.

DPPH Radical Scavenging	Leaf Oil	Twig Oil	Quercetin
EC_50_ (µg/mL)	919 ± 64.4 ^a^	814 ± 42.5 ^b^	13.9 ± 0.16 ^c^

Note: Mean values followed by different letters within the table are statistically different at the 0.05 significance level (*n* = 3). Statistical analysis was performed using the software tool IRRISTAT ver. 5.0 (International Rice Research Institute, Laguna, Philippines).

**Table 5:** Main constituents and bioactivities of essential oils from some *Glycosmis* species.

N^o^	Origin of Samples	Species	Plant Part	Essential Oil Concentration	Main Compounds	Bioactivities	References
1	Vietnam	*G. lanceolata*	Leaves	0.35% (DWB)	Limonene (59.3%), β-caryophyllene (8.8%)	NA	[28]
2	China	*G. lucida*	Leaves	0.35% (DWB)	Elixene (19.81%), spathulenol (10.68%), anethole (12.05%), verbenone (10.32%)	Repellent activity against insects	[18]
3	Vietnam	*G. mauritiana*	Leaves	0.3% (FWB)	Myristicin (21.28%), (Z)-13-docosenamide (9.07%)	NA	[20]
			Twigs	0.2% (FWB)	Myristicin (17.25%), (Z)-13-docosenamide (13.41%)	NA	
4	China	*G. parviflora*	Aerial parts	0.64% (DWB)	(Z)-Caryophyllene (20.6%), methyl isoeugenol (11.1%)	Toxicity against insects	[19]
5	India	*G. pentaphylla*	Bark	0.05% (FWB)	2-Undecanone (58.1%), 2-tridecanone (23.4%)	NA	[15]
			Leaves	0.025% (FWB)	2-Tridecanone (36.8%), 6,10,14-trimethyl-2-pentadecanone (13.1%), hexadecanoic (palmitic) acid (25.6%)	NA	
			Seeds	0.2% (FWB)	Linalool (24.7%), terpinen-4-ol (19.2%)	NA	
6	India	*G. pentaphylla*	Leaves	0.1% (FWB)	Benzaldehyde oxime * (15.66%), bicyclo [6.1.0] non-1-ene * (18.93%)	Larvicidal activity; antioxidant activity (IC_50_ = 21.92 µg/mL)	[17,29]
7	India	*G. pentaphylla*	Leaves	NA	Phytol (28.03%), bicyclo[5.2.0]nonane, 2-methylene-4,8,8-trimethyl-4-vinyl- (10.93%) *, 1,19- eicosadiene (9.84%)	NA	[16]
8	Vietnam	*G. pentaphylla*	Leaves	0.0016% (FWB)	β-Ocimene (23.10%) **, caryophyllene ** (16.14%)	NA	[21]
9	Vietnam	*G. puberula* var. *eberhardtii*	Leaves	0.15% (FWB)	Linalool (12.2%), (*E*)-*β*-caryophyllene (25.1%), α-humulene (28.3%)	Herbicidal activity	[22]

Note: FWB = calculated based on fresh weight; DWB = calculated based on dry weight; NA = not available; * compound is not found in the Dictionary of Natural Products; ** the elution orders are not correct in that literature.

## Data Availability

All data are available in this publication.

## Supplementary Materials

The following supporting information can be downloaded at: https://www.mdpi.com/article/10.3390/molecules31132246/s1, Figure S1. The chromatogram of essential oil from leaves of wild-growing *Glycosmis lanceolata* in Vietnam; Figure S2. The chromatogram of essential oil from twigs of wild-growing *Glycosmis lanceolata* in Vietnam.

## Abbreviations

The following abbreviations are used in this manuscript:

CO_2_	Carbon dioxide
DMEM	Dulbecco’s Modified Eagle Medium
DPPH	1,1-diphenyl-2-picrylhydrazyl
DWB	Calculated based on dry weight
EC_50_	Half-maximal effect concentration
FBS	Fetal bovine serum
FWB	Calculated based on fresh weight
IC_50_	Half-maximal inhibitory concentration
iNOS	Inducible nitric oxide synthase
LPSs	Lipopolysaccharides
L-NMMA	N^G^-methyl-L-arginine acetate
MIC	Minimum inhibitory concentration
MTT	3-(4,5-dimethylthiazol-2-yl)-2,5-diphenyl-tetrazolium bromide
NA	Not available
NO	Nitric oxide
NO_2_^−^	Nitrite
ROS	Reactive oxygen species
NaNO_2_	Sodium nitrite
Tr	Trace
UD	Undetermined
