# Supplementary material for: GC-MS Profiling and In Vitro Assessment of Antimicrobial, Antioxidant, and Anti-Inflammatory Activities of Essential Oils from Wild-Growing Glycosmis lanceolata (Blume) D. Dietr. in Vietnam
Source: Molecules. 2026 Jun 25;31(13):2246. doi: 10.3390/molecules31132246 (PMC13363421; doi:10.3390/molecules31132246)
Supplement: Supplementary file 1 [file molecules-31-02246-s001.zip › molecules-4362168-supplementary.pdf]

molecules

MDPI

Supplementary materials

GC-MS profiling and *in vitro* assessment of antimicrobial, antioxidant, and anti-inflammatory activities of essential oils from wild-growing *Glycosmis lanceolata* (Blume) D. Dietr. in Vietnam

Quang Vuong Le [1], Ha Thi Thu Chu [2,*], Thuy Thi Thu Dinh [3], Thi Minh Chau Dao [1], Thi Huyen Thai [4], Thi Nghiem Vu [5], Ha Chi Vuong [6], and William N. Setzer [7,8]

[1] Biology Faculty, Vinh University, 182 Truong Vinh, Nghe An Province 43000, Vietnam; vuong201173@vinhuni.edu.vn (Q.V.L.); daochau27@gmail.com (T.M.C.D)
[2] Institute of Biology, Vietnam Academy of Science and Technology (VAST), 18 Hoang Quoc Viet, Nghia Do, Ha Noi 10072, Vietnam
[3] Institute of Chemistry, Vietnam Academy of Science and Technology, 18 Hoang Quoc Viet, Nghia Do, Ha Noi 10072, Vietnam; dttthuy@ich.vast.vn
[4] Faculty of Agronomy, University of Agriculture and Forestry (Huaf), Hue University, 102 Phung Hung, Hue City 49000, Vietnam; thaihuyen@hueuni.edu.vn
[5] Institute of Materials Science, Vietnam Academy of Science and Technology, 18 Hoang Quoc Viet, Nghia Do, Ha Noi 10072, Vietnam; vtnghiem@ims.vast.ac.vn
[6] Newton Grammar School, 136 Ho Tung Mau, Phu Dien ward, Hanoi 10053, Vietnam; vuonghachi2203@gmail.com
[7] Aromatic Plant Research Center, 230 N 1200 E, Suite 100, Lehi, UT 84043, USA; setzerw@uah.edu
[8] Department of Chemistry, University of Alabama in Huntsville, Huntsville, AL 35899, USA

* Correspondence: cttha@ib.vast.vn (H.T.T.C.)

Figure S1. The chromatogram of essential oil from leaves of wild-growing *Glycosmis lanceolata* in Vietnam — Page 2

Figure S2. The chromatogram of essential oil from twigs of wild-growing *Glycosmis lanceolata* in Vietnam — Page 2

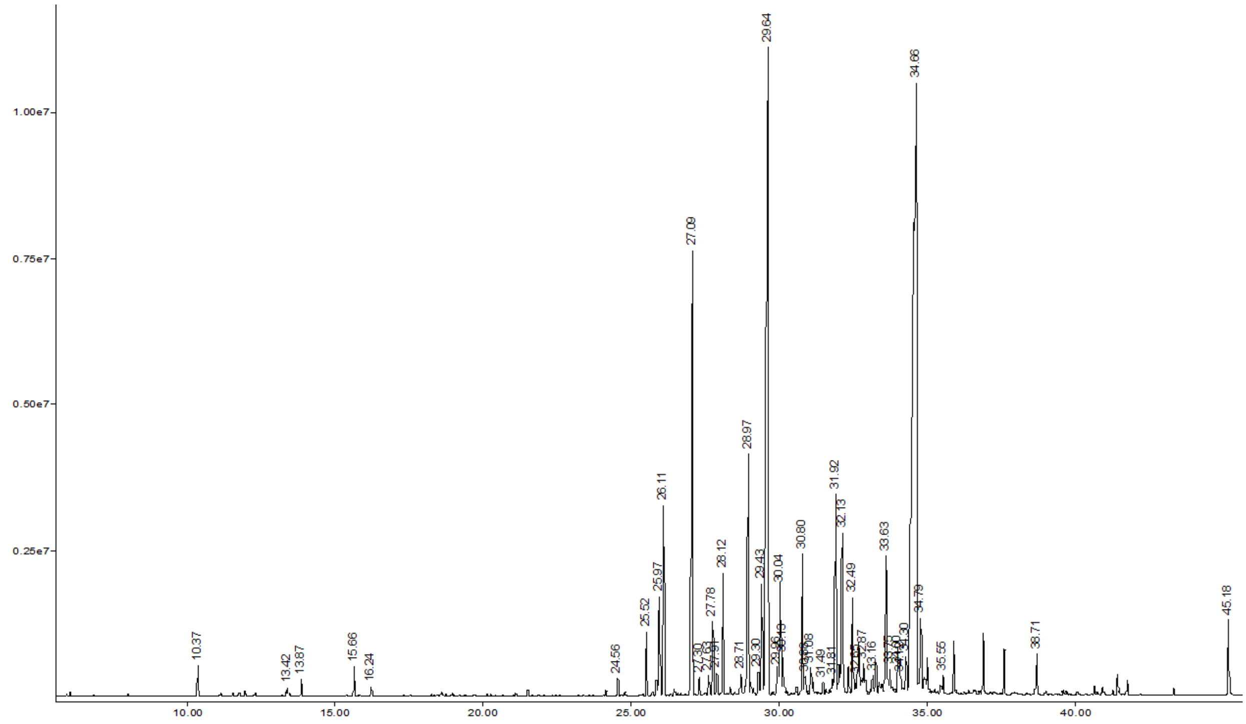

Figure S1. The chromatogram of essential oil from leaves of wild-growing *Glycosmis lanceolata* in Vietnam

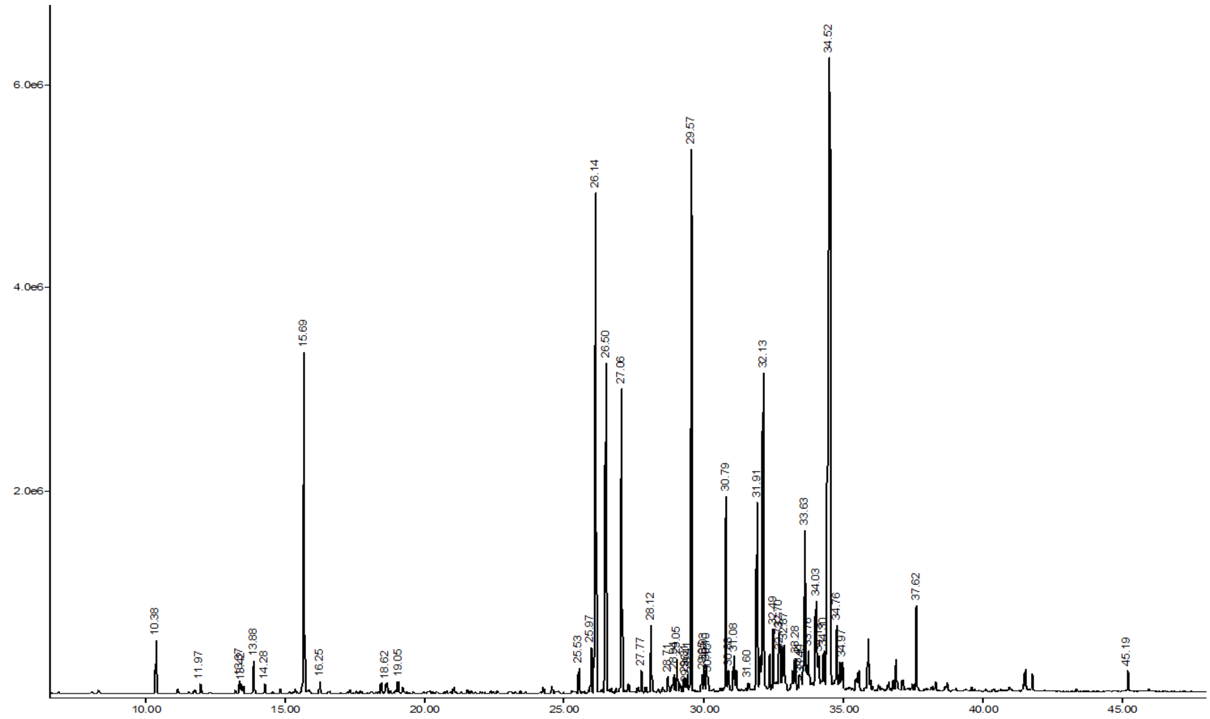

Figure S2. The chromatogram of essential oil from twigs of wild-growing *Glycosmis lanceolata* in Vietnam
